# DNA Qualification Workflow for Next Generation Sequencing of Histopathological Samples

**DOI:** 10.1371/journal.pone.0062692

**Published:** 2013-06-06

**Authors:** Michele Simbolo, Marisa Gottardi, Vincenzo Corbo, Matteo Fassan, Andrea Mafficini, Giorgio Malpeli, Rita T. Lawlor, Aldo Scarpa

**Affiliations:** 1 ARC-NET Research Centre, University of Verona, Verona, Italy; 2 Department of Pathology and Diagnostics, University of Verona, Verona, Italy; 3 Department of Surgery and Oncology, Azienda Ospedaliero-Universitaria Integrata di Verona, Verona, Italy; Deutsches Krebsforschungszentrum, Germany

## Abstract

Histopathological samples are a treasure-trove of DNA for clinical research. However, the quality of DNA can vary depending on the source or extraction method applied. Thus a standardized and cost-effective workflow for the qualification of DNA preparations is essential to guarantee interlaboratory reproducible results. The qualification process consists of the quantification of double strand DNA (dsDNA) and the assessment of its suitability for downstream applications, such as high-throughput next-generation sequencing. We tested the two most frequently used instrumentations to define their role in this process: NanoDrop, based on UV spectroscopy, and Qubit 2.0, which uses fluorochromes specifically binding dsDNA. Quantitative PCR (qPCR) was used as the reference technique as it simultaneously assesses DNA concentration and suitability for PCR amplification. We used 17 genomic DNAs from 6 fresh-frozen (FF) tissues, 6 formalin-fixed paraffin-embedded (FFPE) tissues, 3 cell lines, and 2 commercial preparations. Intra- and inter-operator variability was negligible, and intra-methodology variability was minimal, while consistent inter-methodology divergences were observed. In fact, NanoDrop measured DNA concentrations higher than Qubit and its consistency with dsDNA quantification by qPCR was limited to high molecular weight DNA from FF samples and cell lines, where total DNA and dsDNA quantity virtually coincide. In partially degraded DNA from FFPE samples, only Qubit proved highly reproducible and consistent with qPCR measurements. Multiplex PCR amplifying 191 regions of 46 cancer-related genes was designated the downstream application, using 40 ng dsDNA from FFPE samples calculated by Qubit. All but one sample produced amplicon libraries suitable for next-generation sequencing. NanoDrop UV-spectrum verified contamination of the unsuccessful sample. In conclusion, as qPCR has high costs and is labor intensive, an alternative effective standard workflow for qualification of DNA preparations should include the sequential combination of NanoDrop and Qubit to assess the purity and quantity of dsDNA, respectively.

## Introduction

A standardized and cost-effective workflow for the qualification of DNA preparations is essential to assure interlaboratory reproducible results, whatever the DNA source or extraction method applied. DNA qualification consists of both the quantification of double strand DNA (dsDNA) and the assessment of its suitability for downstream applications. Its reliability is particularly relevant in the advent of next-generation sequencing (NGS) technologies: these hold the key to identifying the compendium of genetic alterations that specifically occur in human diseases, such as cancer [1]–[4], and provide for potential clinical applications. Indeed, NGS has made it possible to examine millions of sequences using less DNA per assay than in the past decades.

While NGS sequencers yield results never achieved before, the workflow is a lengthy, expensive, labor-intensive process that further underlines the need for the appropriate management of the input material. Non-standardized DNA qualification can negatively impact sequencing performances [5], resulting in a waste of samples, low confidence results and higher costs. Inconsistency in DNA qualification among laboratories is common experience, that is heightened in collaborative projects. These require the collection of DNA samples from different centers that may not use the same procedures to qualify DNA.

Partially degraded DNA from formalin-fixed paraffin-embedded [FFPE] tissues is already used for diagnostic applications but may also be used in NGS [6]. The potential of using samples processed within standard clinical diagnostics, however, raises additional technical issues due to the wide variety of tissue processing and storage procedures, that significantly affect DNA yield and quality [7]–[11] and the small amounts of tissue available.

There are three most common techniques for nucleic acids quantification: UV spectrophotometry [12] using NanoDrop instrument (Thermo Scientific, Wilmington, MA) [13]; dsDNA-specific fluorimetry using Qubit (Life Technologies, Grand Island, NY); quantitative PCR (qPCR) that simultaneously assesses DNA quantity and suitability for PCR amplification.

Although a number of studies have compared different DNA extraction and qualification methods, each one only partially covered the topic of DNA qualification, concentrating mainly on the extraction step [11], [14]–[17]. When the topic of DNA qualification was addressed, contradictory results emerged depending on the source of DNA being analyzed. For instance, Foley *et al.*, analyzed DNA from fresh bovine samples reporting that NanoDrop and Qubit measurements were “generally consistent” [11], while Haque *et al*. analyzed DNA from cell lines and concluded that UV spectrophotometry was the most concordant and precise DNA quantification method [5]. O'Neill *et al.* and Sironen *et al.*, respectively analyzing human and boar fresh samples, found that NanoDrop heavily overestimates DNA concentration [16], [17]. The topic of assessing DNA purity by reading 260/230 nm and 260/280 nm, as well as the comparison with qPCR, was also treated by some of the cited publications, but a definite workflow was never apparent. Moreover, studies involving the qualification of DNA from pathological samples, especially FFPE ones, are lacking.

Therefore, in the present work we compared these common technologies to define the minimum essential workflow for rapid, efficient and cost-effective DNA qualification for NGS on histopathological samples. Variables considered as possibly influencing estimates were: (i) operator-dependent variability; (ii) nucleic acid concentration; (iii) RNA contamination; (iv) quality of DNA preparations, intended as quantity of dsDNA and suitability for downstream applications.

## Materials and Methods

### Samples

A series of 17 human genomic DNA samples were used, comprising two commercial preparations and 15 from cells and tissues. The two commercial human genomic DNA preparations of known concentrations were: L, 200 ng/µl (Universal unmethylated DNA, Chemicon Int., Billerica, MA); and G, 5 ng/µl (PrimerDesign Ltd., UK). Human tissues and cancer cell lines came from the ARC-NET biobank at University of Verona, Verona, Italy (**Table 1**) and comprised: i) 6 fresh frozen tissues (five pancreas and one spleen); ii) 6 FFPE tissues (four pancreas, one duodenum and one liver); iii) 3 adenocarcinoma cell lines (GER, PC2, and PC3) [18].

**Table 1:** Human genomic DNA preparations used in the study.

*Sample ID*	*Source* a	*Tissue of origin*	*Integrity by gel electrophoresis* b
L	Commercial preparation	not declared	HMW
G	Commercial preparation	not declared	HMW
GER	Cell line	pancreas	HMW
PC2	Cell line	pancreas	HMW
PC3	Cell line	pancreas	HMW
FF R	FF tissue	pancreas	HMW
FF A	FF tissue	normal spleen	HMW
FF 46	FF tissue	pancreas	partially degraded
FF 107	FF tissue	pancreas	HMW
FF 148	FF tissue	pancreas	HMW
FF 4N1	FF tissue	pancreas	HMW
FFPE D	FFPE tissue	duodenum	n.a.
FFPE P	FFPE tissue	pancreas	n.a.
FFPE A	FFPE tissue	liver	n.a.
FFPE 3	FFPE tissue	pancreas	n.a.
FFPE 5	FFPE tissue	pancreas	n.a.
FFPE 8	FFPE tissue	pancreas	n.a.

a FF, fresh frozen; FFPE, formalin-fixed paraffin embedded;

b HMW, high molecular weight DNA; n.a., not applicable, as DNA from formalin fixed tissue is always partially degraded and no information is obtained by gel electrophoresis.

### Ethics statement

The materials used have been collected under Program 853 protocol 298CE 15/02/02 and Program 1885 protocol 52438 on 23/11/2010. The protocols include informed consent of the patient and were approved by the local ethics committee of the Integrated Unversity Hospital Trust of Verona. The first approval (prog. 853) regarded the collection of pancreas samples for use in molecular research studies. This was later updated (prog. 1885) for the creation of a coordinated biobank for the collection of samples from all cancer patients that included neoplastic and associated local and distant normal tissue. The approved programs include tissue processing and storage methods of snap frozen tissues stored at −80°C and FFPE of both neoplastic and normal tissue. The latter program included amendments to address the later regulatory issues of data disclosure in genomic studies. Tissues were collected following informed consent of the patients.

### DNA preparation

Genomic DNA and total RNA (the latter only from fresh-frozen samples) was obtained using different extraction kits from Qiagen (Milan, Italy) according to the type of material. The QIAmp AllPrep DNA/RNA mini kit was used for frozen samples and cell lines, while the QIAmp DNA FFPE tissue kit was used for FFPE samples.

### Quantification and qualification of DNA samples by NanoDrop and Qubit

DNA quantity and quality was measured by reading the whole absorption spectrum (220–750 nm) with NanoDrop and calculating DNA concentration and absorbance ratio at both 260/280 and 230/260 nm [19]. NanoDrop ND-2000 is a spectrophotometer that uses two optical fibers installed in the pedestal (emitting light from a Xenon lamp) and a sample arm (spectrometer with linear charge-coupled device [CCD] array). Samples of 1 µl volume are measured without the need for cuvettes or capillaries. The machine was calibrated and cleaned according to the NanoDrop 2000–2000c & 1000 Calibration Check procedure.

Each sample was then quantified with the Qubit fluorometer. This is a quantitation system relying on dyes that only fluoresce when bound to specific molecules, such as dsDNA, ssDNA or RNA. The instrument was calibrated with the Quant-iT dsDNA HS Assay (declared assay range between 0.2–100 ng; sample starting concentration between 10 pg/µl–100 ng/µl) and the Quant-iT dsDNA BR Assay (declared assay range between 2–1000 ng; sample starting concentration between 100 pg/µl and µg/µl), according to the manufacturer's instructions.

### DNA quality assessment by electrophoresis

The extracted DNA was evaluated by loading 100 ng of DNA based on both NanoDrop and Qubit measurements on a 0.8% agarose gel electrophoresis; ethidium bromide stained gels were digitally acquired with a Fluor-S imager equipped with an UV lamp and a 520 nm low-pass filter. Densitometric analysis of gel lanes was executed with the ImageJ software, using sample L as a reference for signal normalization [20].

### DNA quantitation by real-time qPCR

The Genomic DNA Quantification Assay from PrimerDesign is a quantitative PCR developed to detect a single copy region of non-transcribed DNA to avoid false positive signals from possible contamination (e.g. cDNA). Standard curve to measure samples concentrations was constructed in the range from 5 pg/µl to 5,000 pg/µl and the reaction mixture was assembled following the manufacturer's protocol.

### Construction of libraries for next generation sequencing

Forty nanograms of DNA from FFPE samples, as quantified by Qubit, were used for multiplex PCR amplification of 191 amplicons exploring selected regions of a panel of 46 cancer-related genes (Ion AmpliSeq Cancer Panel, Life Technologies, Milan, Italy), according to the manufacturer's protocol. The quality of the libraries obtained was assessed by the Agilent 2100 Bioanalyzer on-chip electrophoresis (Agilent Technologies, Inc; Santa Clara, CA).

### Statistical analysis

Statistical analysis for testing the technologies performances in DNA quantification was based on multiple independent measurements (*n* = 20 for commercial DNA, *n* = 5 for routine samples DNA) taken with both quantitation platforms for each sample. Paired *t*-test was applied to test inter-operator consistency. The Wilcoxon signed rank test was used to compare quantification of DNA against the expected value; the Mann-Whitney test was used to compare the quantification of commercial DNA measured by NanoDrop vs. Qubit. Linear regression was used to compare different dilutions of the DNA samples, considering each measurement (n = 10 for each dilution of commercial samples, n = 5 for each dilution of the tissue samples) as an individual point. R^2^ coefficient of determination and the F-test were used to describe regression line fitness. Bland-Altman analysis was used to display the ratio between NanoDrop or Qubit quantification and the qPCR-derived expected values; average ratio and standard deviation (SD) were used for comparison. One-way ANOVA (with Dunnet's post-hoc test) was used to analyze the influence of RNA contamination on DNA quantification; data were log-transformed before ANOVA to satisfy the equal variances assumption. A p-value <0.05 was regarded as significant. All analyses were performed using GraphPad Prism version 5.00 for Windows (GraphPad Software, San Diego, CA, USA; www.graphpad.com).

## Results

### Sample handling and intra-procedure variability

**Figure 1:**
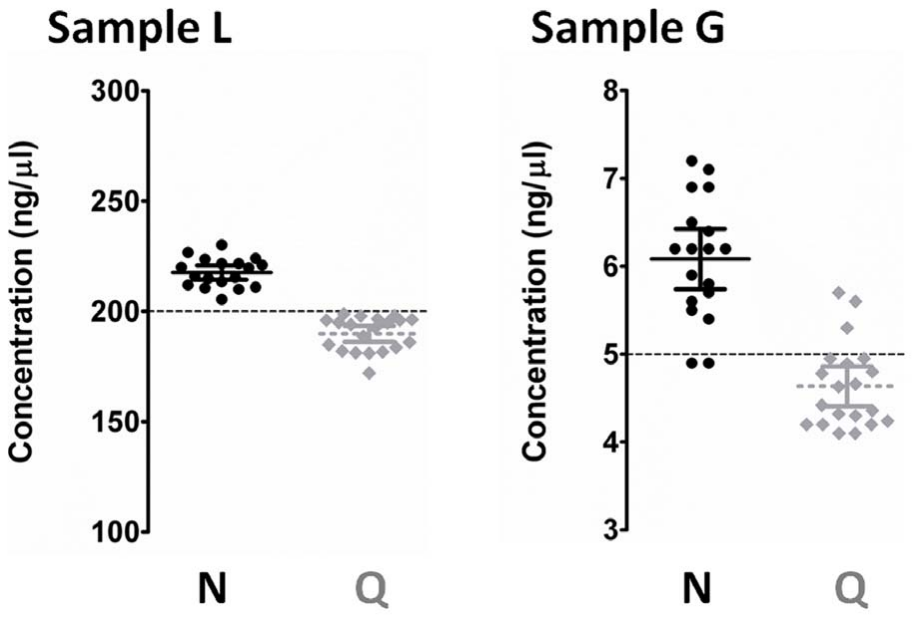
Intra- and inter-method accuracy and precision. Distribution of DNA sample concentration (dispersion chart) was estimated by both NanoDrop (black) and Qubit (gray) on repeated (n = 20) measurements of two commercial human genomic DNA preparations (Sample L 200 ng/µl; Sample G 5 ng/µl). For both samples, NanoDrop overestimated the DNA concentration (+8.8% for L and +24.0% for G, p<0.0003), while Qubit underestimated it (−5.0% for L and −7.3% for G, p<0.005).

In order to first investigate the operator-dependent variability associated with both NanoDrop and Qubit measurements, the two commercial DNA preparations L [200 ng/µl] and G [5 ng/µl]) were tested. DNA concentration was estimated by both NanoDrop and Qubit on repeated (n = 20) measurements. Sample handling intra-operator variability, as measured by a coefficient of variation, was less than 6% for sample L and 14% for the more diluted sample G. Inter-operator consistency was assessed on repeated (n = 10) measurements and showed no significant differences (p = 0.154). For both samples, NanoDrop estimated a DNA concentration higher than Qubit (Mann-Whitney test p<0.0005 for both samples; **Figure 1**).

To test intra-procedure variability a regression line was fitted using 14 dilution points (expected concentration ranging from 200 to 0.5 ng/µl) of sample L, with 10 repeated measurements for each dilution and quantitation platform. The concordance between measured and expected values (slope and intercept of the regression line) was high for both technologies and the average internal error of the regression lines was negligible (R^2^ = 0.99, F-test p<0.0001 for both NanoDrop and Qubit; **figure S1**). However, NanoDrop values were slightly higher than expected (ratio  = 1.06; 95%CI 1.04–1.07) while the opposite was the case for Qubit (measured/expected ratio  = 0.97; 95%CI 0.96–0.98). Moreover, the diluted DNA at 0.5 ng/µl gave no meaningful readings for NanoDrop, while Qubit was able to correctly quantify this sample (mean 0.53 ng/µl; 95%CI 0.47–0.60 ng/µl, n = 10). Finally, the intercept of the regression curve was significantly non-zero for NanoDrop (measured value at theoretical zero concentration: 3.09 ng/µl, 95%CI 1.9–4.3), consistent with the NanoDrop declared limit of detection of 2 ng/µl.

### DNA quantification by NanoDrop, Qubit and qPCR

**Figure 2:**
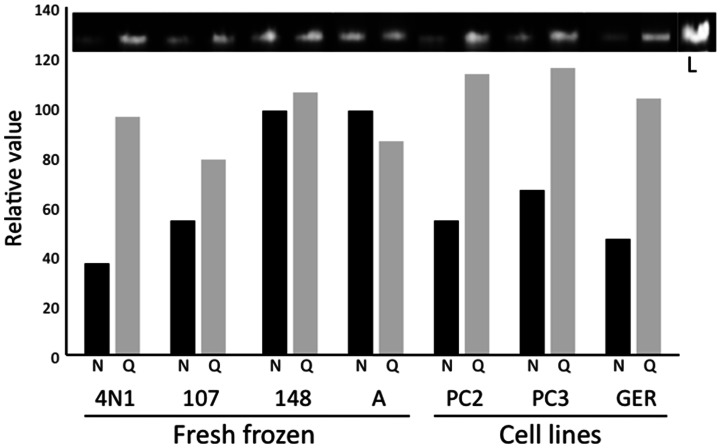
Significant discrepancies in DNA quantification by NanoDrop and Qubit. A total of 100 ng of DNA based on NanoDrop (N, black bars) or Qubit (Q, grey bars) measurements was analyzed by electrophoresis on 0.8% agarose gel. Sample ID is indicated at the bottom. Lane L contains 200 ng of DNA as the reference for normalization. Densitometric analysis (bar chart) was performed by ImageJ software [20]. It is clear from the electrophoretic bands and their densitometric charts that NanoDrop overestimates DNA concentration.

NanoDrop concentration values were higher than Qubit for all samples but sample A (**Table 2**). To confirm this observation and assay DNA quality, 100 ng of DNA based on either NanoDrop or Qubit measurements were loaded side by side on a 0.8% agarose gel (**Figure 2**). All DNAs showed satisfactory DNA integrity without evident smears, with the exception of sample FF46 (not shown). Densitometric analysis confirmed that NanoDrop overestimated the DNA content of most samples (Figure 2), while Qubit measures were consistent with the densitometric evaluation of agarose gel bands of integer DNA.

**Table 2:** Comparison of sample concentration according to NanoDrop and Qubit quantitation platforms by samples dilutions.

*Sample*	*NanoDrop*				*Qubit*			
	*Obs 1:1*	*Obs 1:5*	*Obs 1:10*	*R^2^*	*Obs 1:1*	*Obs 1:5*	*Obs 1:10*	*R^2^*
GER	29.9 (±2.2)	3.8 (±0.7)	2.2 (±0.4)	0.991	14.4 (±0.14)	2.7 (±0.4)	1.0 (±0.04)	0.998
PC2	86.7 (±5.9)	24.7 (±6.6)	8.8 (±0.8)	0.979	37.4 (±0.63)	11.8 (±0.48)	5.7 (±0.8)	0.992
PC3	44.1 (±3.5)	15.7 (±0.7)	7.0 (±0.4)	0.995	31.0 (±0.67)	6.0 (±0.3)	3.5 (±1.2)	0.983
FF R	510 (±20.9)	149 (±23.9)	62.8 (±17.5)	0.973	134 (±18.1)	63.0 (±21.5)	44.2 (±19.4)	0.961
FF A	30.6 (±3.7)	6.6 (±0.9)	2.8 (±0.2)	0.983	37.2 (±0.36)	4.3 (±0.3)	2.8 (±0.02)	0.996
FF 46	52.3 (±3.2)	9.2 (±1.6)	3.3 (±1.4)	0.995	10.2 (±1.6)	1.8 (±0.3)	1.0 (±0.05)	0.972
FF 107	119.5 (±3.2)	18.9 (±9.4)	13.9 (±0.7)	0.989	64.5 (±15.0)	11.0 (±0.4)	5.7 (±0.5)	0.947
FF 148	98.2 (±0.6)	12.5 (±10.3)	9.8 (±2.9)	0.983	88.8 (±15.2)	14.5 (±3.1)	8.4 (±0.09)	0.967
FF 4N1	293.0 (±3.0)	53.7 (±13.3)	33.1 (±2.2)	0.997	88.1 (±4.45)	21.3 (±5.5)	8.7 (±0.6)	0.990
FFPE D	160.3 (±1.4)	30.3 (±0.9)	15.9 (±0.9)	0.999	20.4 (±4.4)	3.2 (±0.6)	2.0 (±0.6)	0.950
FFPE P	50.8 (±3.7)	7.9 (±3.6)	3.5 (±2.2)	0.988	9.0 (±2.2)	2.0 (±0.8)	0.8 (±0.2)	0.950
FFPE A	93.8 (±2.1)	24.2 (±1.8)	10.3 (±0.78)	0.993	4.3 (±0.4)	0.5 (±0.04)	0.3 (±0.1)	0.984

**Note:** Obs, observed concentration: mean values (±95% Confidence Interval) of 5 observations per measurement point at 1∶1, 1∶5 and 1∶10 dilutions. R^2^, coefficient of determination. F-test for significance of the analysis returned a p<0.0001 for all samples.

To test intra-procedure variability, three dilutions (1∶1, 1∶5 and 1∶10) of each sample were measured 5 times. The concordance between measured values and dilution was high for both technologies with a correlation coefficient always ≥0.95 (Table 2).

**Figure 3:**
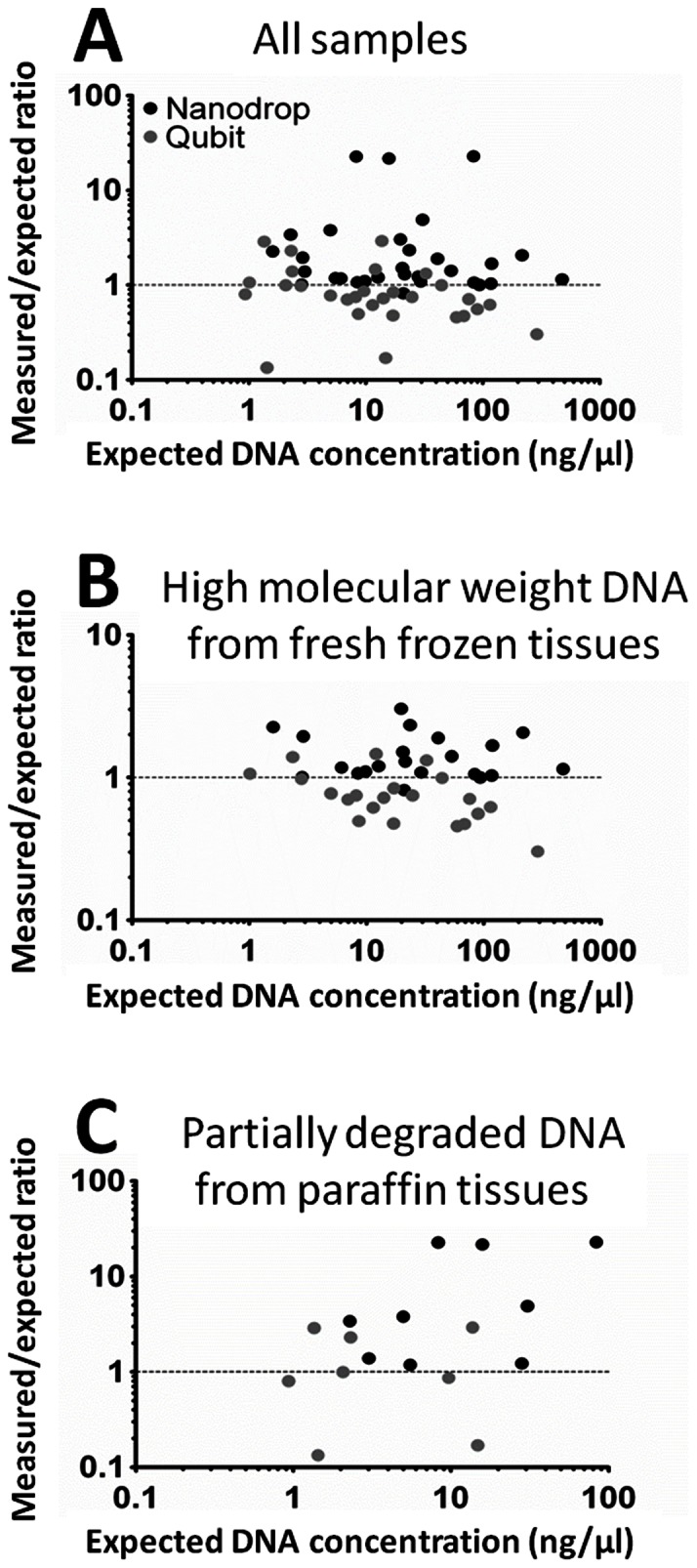
Cross-validation of DNA samples quantification by qPCR. Bland-Altman plots for inter-technology (NanoDrop or Qubit vs. qPCR) comparison of all samples (A), and according to the different sample sources, as indicated (B, C). A) Qubit measurements show high correlation (mean measured/expected ratio  = 0.92; SD  = 0.69; Wilcoxon signed rank test p = 0.07) with the measurements obtained by qPCR (x-axis), whereas NanoDrop measurements tend to overestimate samples concentration (mean measured/expected ratio  = 3.8; SD  = 6.4; Wilcoxon signed rank test p<0.0001). B) Fresh frozen sample quantification by NanoDrop overestimates (mean measured/expected ratio  = 1.48; SD  = 0.57; Wilcoxon signed rank test p<0.01) the DNA concentration detected by quantitative PCR, while Qubit underestimates (mean measured/expected ratio  = 0.78; SD  = 0.32; Wilcoxon signed rank test p<0.001) the value. C) In formalin-fixed paraffin-embedded samples a better concentration estimation is obtained by Qubit (mean measured/expected ratio  = 1.23; SD  = 1.15; Wilcoxon signed rank test p = 0.91) than by NanoDrop (mean measured/expected ratio  = 9.21; SD  = 9.95; Wilcoxon signed rank test p<0.004).

To compare the accuracy of the two methods in quantifying DNA, a PCR-based assay (PrimerDesign) was used as reference, as it both quantitates DNA and assesses its suitability for PCR amplification. NanoDrop and Qubit measurements of three dilutions of samples (1∶1, 1∶5, 1∶10) were plotted against the concentrations as detected by the qPCR assay. From the Bland-Altman analysis for inter-technology comparison, NanoDrop data showed a high dispersion and a higher content of DNA compared to qPCR (mean measured/expected ratio  = 3.8; SD  = 6.4, **Figure 3**); Qubit measurements showed high concordance with qPCR data (mean measured/expected ratio  = 0.92; SD  = 0.69).

To evaluate the influence of DNA quality on measurements, samples estimations were grouped based on their characteristics (Figure 3). Bland-Altman analysis showed similar performance of both NanoDrop and Qubit on high molecular weight DNA from frozen samples and cell lines, while Qubit had better performance for FFPE samples (NanoDrop measured/expected ratio = 9.21, Wilcoxon signed rank test p<0.004; Qubit measured/expected ratio  = 1.23, Wilcoxon signed rank test p = 0.91).

### RNA contamination influences DNA quantification

**Figure 4:**
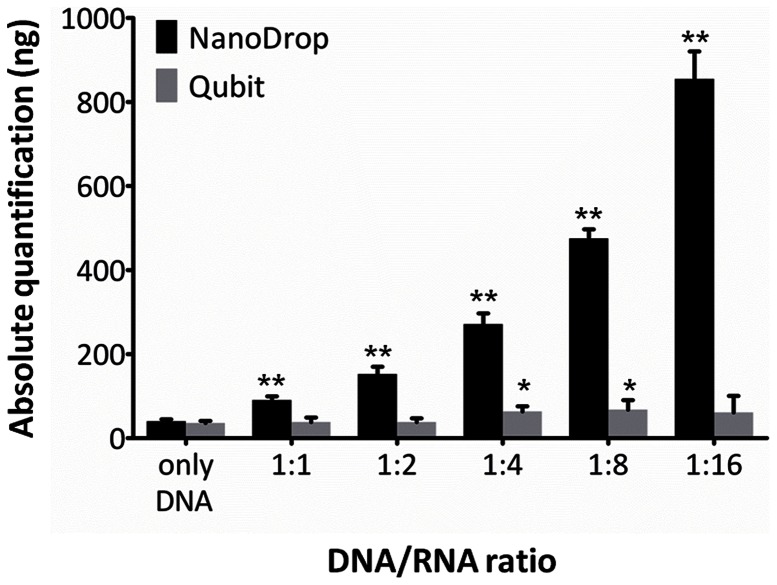
Influence of RNA contamination on DNA quantification. DNA quantifications (n = 5) by NanoDrop and Qubit in the presence of RNA contamination. A DNA sample with a concentration of 38 ng/µl was mixed with different volumes of total RNA at 33 ng/µl extracted from the same tissue sample to obtain the indicated ratios; bars and brackets indicate mean and 95% confidence interval; asterisks show measurements significantly different from pure DNA (* p<0.05; ** p<0.001; Dunnett's post-hoc test). NanoDrop measurements were heavily influenced by the presence of RNA contamination (ANOVA p<0.0001), whereas Qubit values were less affected (ANOVA p<0.01). Black bars  =  NanoDrop; gray bars  =  Qubit.

To estimate the influence of RNA contamination on DNA quantification, genomic DNA (sample 148) at a concentration of 38 ng/µl was mixed with different volumes of total RNA at a concentration of 33 ng/µl, extracted from the same tissue sample, to obtain DNA/RNA ratios of 1∶1, 1∶2, 1∶4, 1∶8, and 1∶16 (**Figure 4**).

NanoDrop detected the presence of RNA contamination, showing increased values of A_260_ reads, and as such impairing the appropriate measurement of the dsDNA concentration already at 1∶1 DNA:RNA ratio (p<0.001). Moreover, increasing RNA contamination led to the concomitant increase of the A_260/280_ ratio, from 1.85 of the sample with no RNA contamination up to 2.0 for the sample displaying severe RNA contamination (data not shown). Qubit was much less influenced by RNA contaminations, with measured DNA concentration significantly increasing only when the DNA:RNA ratio was 1∶4 or beyond (p<0.05).

### Cost and time comparison

A direct comparison of the costs and labor involved in DNA quantification by NanoDrop, Qubit, and qPCR is reported in **Table 3**. NanoDrop is the fastest and most inexpensive approach for DNA quantification with a sample processing time of less than one minute and a sample cost of ∼0.10€. Qubit is the less expensive platform, but has a sample processing time of over 5 minutes and a sample cost of ∼0.53€. The qPCR-based method is the most expensive both in terms of platform and sample costs of ∼1.80€, and is the most time intensive on limited numbers of samples (operator time requirements is limited to about 20 minutes, but the total process goes on more than 2 hours). It should be mentioned that a multi-well qPCR approach might be most suitable to high output requirements (i.e., extensive biobanking, international multi-institutional consortia).

**Table 3:** Comparison of estimated costs and time requirements per sample for DNA quantification using NanoDrop, Qubit, or qPCR.

	NanoDrop	Qubit	qPCR
*Cost analysis*			
Sample preparation	0.10 €	0.10 €	0.10 €
Instrument consumables	0.00 €	0.33 €	1.80 €
Plastic materials	0.00 €	0.10 €	0.10 €
**Total direct costs**	**∼0.10 €**	**∼0.53 €**	**∼2.00 €**
Hardware & software costs	**∼**12,000.00 €	**∼**1,400.00 €	**∼**35,000.00 €*
*Time requirements*			
Sample/instrument preparation	10 sec	5 min	5 min
Run time and results analysis	20 sec	20 sec	2 h
**Total time**	**30 sec**	**5 min 20 sec**	**2 h 05 min**

* indicative cost for a standard qPCR instrument.

### DNA qualification for next-generation-sequencing library construction

To evaluate the effect of DNA quantification on NGS workflow, the construction of NGS libraries (Ion Torrent Ampliseq Cancer Panel) was performed using DNA from FFPE sample P. Forty nanograms of DNA, as calculated by NanoDrop or Qubit, were processed according to the manufacturers' protocol and the quality of the obtained library was evaluated by Agilent on-chip electrophoresis. The NanoDrop-measured sample repeatedly yielded no library amplification (n = 3). Conversely, the Qubit-measured sample gave an adequate library for subsequent sequencing (data not shown).

**Figure 5:**
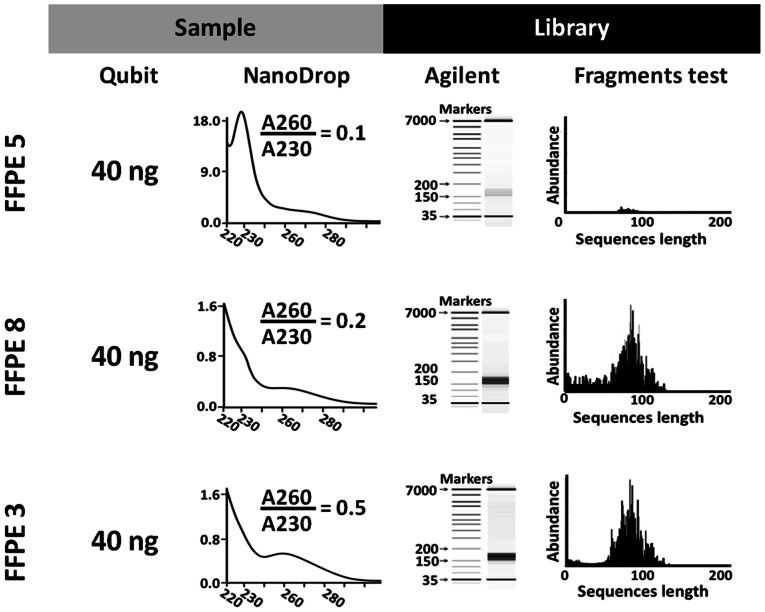
DNA qualification for next-generation sequencing applications. Effect of low-quality DNA on next-generation sequencing (NGS) workflow. Three FFPE samples were tested for construction of NGS amplicon libraries (Ion Torrent Ampliseq Cancer Panel). Qubit: 40 ng of DNA according to Qubit measurement were processed using the Ampliseq library construction kit (multiplex PCR amplification of 191 DNA regions from 46 cancer-related genes). NanoDrop: absorption spectra of samples showed different degrees of organic contamination (230 nm spike, A260/A230 ratio). Agilent: quality and quantity of the obtained libraries were evaluated by Agilent high sensitivity assay on-chip electrophoresis, where the library is represented by the large band between 150 and 200 bp. Fragments test: histogram showing length and abundance of produced sequences. Sample FFPE 5 did not produce a good library due to high organic contamination; this is revealed by the remarkable spike at 230 nm that concurs to the low 260/230 ratio, and explains the faint electrophoretic band and the almost flat fragments test histogram.

The impact of low DNA purity was then assessed on samples FFPE 3, FFPE 5 and FFPE 8 (**Figure 5**): 40 ng of DNA based on Qubit quantification gave adequate libraries for subsequent sequencing in 2 of the 3 samples; only FFPE 5 did not provide a suitable library (0/3 independent runs). The reason for this failure was evident from the NanoDrop spectrum of FFPE 5 that showed a spike at 230 nm and a 260/230 ratio of 0.1, indicating organic contamination.

## Discussion

The pre-analytical steps that need standardization and quality control in order to obtain consistent and inter-laboratory reproducible results from DNA analysis are three: i) preparation and storage conditions of tissue/biological materials; ii) DNA extraction methods; iii) DNA qualification.

The present work deals with the third step, presenting a standardized workflow for DNA qualification, whatever the DNA source or extraction method used. Our data suggest that the sequential use of spectrophotometric and fluorescence-based methodologies permits the cost-effective assessment of DNA quality for high troughput downstream applications.

Although the idea of comparing and evaluating NanoDrop and Qubit is not novel, this topic has not been fully addressed in the literature. Most reports deal with extraction methods (see for example [11], [14], [17]) and only two deal with the DNA qualification step [5], [16]. Both these studies used DNA from fresh materials but reached conflicting conclusions. O'Neill *et al*. [16] favor Qubit while Haque *et* *a*l. [5] claim that spectrophotometry is superior to fluorimetry and qPCR. In synthesis, the issue of a standardized workflow for DNA qualification and its suitability for downstream sophisticated applications, such as NGS, is lacking or only partially approached in the literature. Moreover, to the best of our knowledge, the issue of qualification of DNA from human diagnostic specimens, either fresh-frozen or formalin-fixed paraffin-embedded, is also not satisfactorily addressed in the literature. These samples are the basis of human molecular clinical research but are often available in small amounts. Thus, a good qualification step is mandatory to avoid wasting extremely precious material.

The advent of the next-generation sequencing era has revolutionized translational research and will steadily influence the diagnostic practice in the near future. Using limited amounts of dsDNA, the mutational and methylation status of numerous genes can be evaluated simultaneously, which is critical for the realization of a personalized medicine.

As with all complex procedures, pre-analytic management of specimens is a crucial step for downstream performance. In this setting, different methods of tissues/cells processing and storage can significantly affect the quality and quantity of the extracted DNA. The situation is further complicated when DNA is received from different laboratories. This is a common occurrence in collaborative projects, where DNA quantities show high degrees of discrepancy among different laboratories, mainly due to differing quantification procedures. This causes a waste of sample material for repeated and additional quality control tests, with detrimental effects in terms of cost and time consumption. An adequate workflow for measurement of quantity and quality of DNA for its use in next generation sequencing analysis is currently lacking, yet it represents one of the most remarkable current topics in the field. This is particularly true for histopathological samples, both FFPE and fresh-frozen, as they are often available in very limited amounts.

In this study, the performance of the most widespread DNA quantification methods (NanoDrop, Qubit, and qPCR) was tested on human genomic DNA from different sources, as a comprehensive representation of all sample typologies currently used in molecular diagnostics. The intra- and inter-operator variability was negligible when assessed on 10 repeated measurements performed by two operators. Intra-methodology variability was minimal, when assessed by linear regression on repeated estimates of scalar dilutions, while consistent inter-methodology divergences were observed. In fact, NanoDrop measured DNA concentrations higher than those of Qubit and its consistency with dsDNA quantification by qPCR was limited to high molecular weight DNA extracted from FF samples and cell lines, where total DNA and dsDNA virtually coincide. In partially degraded DNA from FFPE samples, only Qubit results were highly replicable and consistent with qPCR measurements. At variance, NanoDrop heavily overestimated DNA concentration in FFPE samples. As a consequence, histopathological samples quantified by NanoDrop alone gave poor performances in a NGS library set-up. On the other hand, the sole use of Qubit gives no information about DNA organic contamination and this too may result in library failure (Figure 5). Only the combination of both technologies allows the correct qualification of DNA samples for NGS; this combination also enables the detection of impurities, and thus their possible removal from precious samples, available in limited amounts, before they enter the library construction step.

The qPCR method has the advantage of simultaneously providing information about quantity and suitability of DNA samples for downstream PCR applications. This technique, however, does not provide information to explain why a certain samples do not amplify, which is especially important when dealing with clinical case FFPE tissue of very limited amounts. Furthermore, it is less compatible with routine laboratory practice, due to its higher costs and labor intensity, which are much higher than those of both NanoDrop and Qubit platforms.

The influence of RNA contamination on DNA quantification should be taken into account when using protocols for DNA/RNA co-purification. UV-based analyses such as NanoDrop cannot distinguish DNA from RNA molecules [13], [21], [22]. This nondiscriminatory trait can be overcome by the concomitant use of fluorescence-based Qubit technology [12], [23]–[25]; the combination of both systems yields the most complete information to correctly qualify histopathological derived DNA.

## Conclusion

The creation of a standardized DNA quantification procedure needs to be effective regardless of the origin of the DNA and, in particular, must be valid for histopathological samples that are vital to applied research. Our data strongly suggest that the ideal workflow to qualify DNA from histopathological samples as suitable for NGS is to first assess the presence of contaminants in the sample with NanoDrop, and subsequently use Qubit to quantify the dsDNA. The application of qPCR-based methods is unpractical in many scenarios due to relatively high direct costs and labor intensity. The choice should be based on the cost-effectiveness of the workflow, which may differ for a single laboratory performing a limited set of experiments or the quality assurance platform of a large bio-bank.

## Supporting Information

Figure S1 **Concordance between expected concentration of a commercial DNA preparation and measured values by either NanoDrop or Qubit technology (14 points calibration curve analyzed by linear regression, n = 10 per concentration point, R^2^ = 0.99 and p<0.0001 for both regression lines).** A- full calibration curve; B- magnification of the calibration curve at lower concentration points. Brackets illustrate 95% confidence intervals. Parameters of the regression lines were as follows: NanoDrop [measured] = 1.06×[expected]+3.09; Qubit [measured] = 0.97×[expected]−0.61. Intercept was significantly non-zero for NanoDrop only (95% confidence interval of intercept  = 1.9–4.3), consistent with the NanoDrop declared limit of detection of 2 ng/µl.
(TIF)
