# Supplementary figures and images for: DNA Qualification Workflow for Next Generation Sequencing of Histopathological Samples
Source: PLoS One. 2013 Jun 6;8(6):e62692. doi: 10.1371/journal.pone.0062692 (PMC3675123; doi:10.1371/journal.pone.0062692)

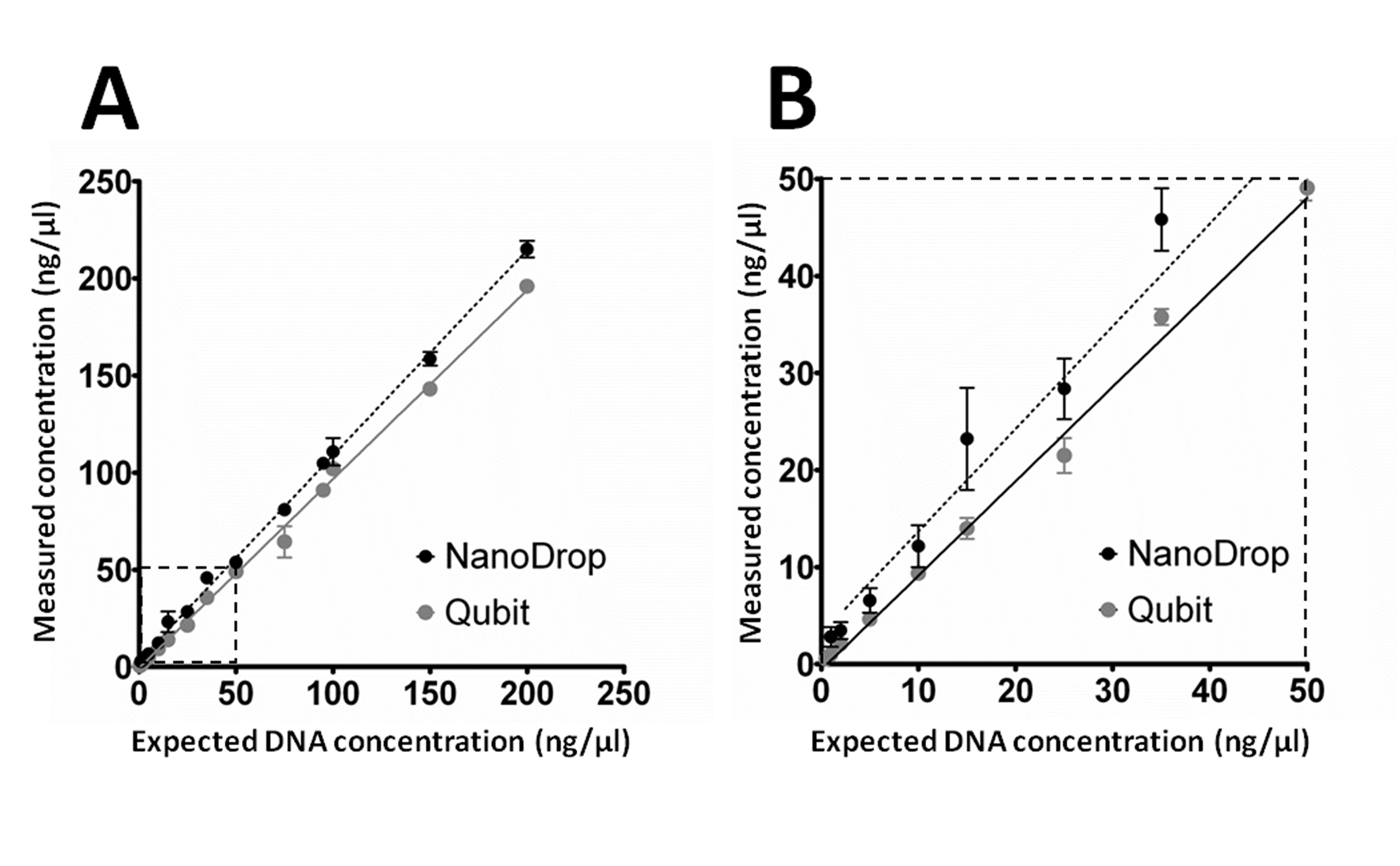

Supplement: Figure S1 — Concordance between expected concentration of a commercial DNA preparation and measured values by either NanoDrop or Qubit technology (14 points calibration curve analyzed by linear regression, n = 10 per concentration point, R2 = 0.99 and p<0.0001 for both regression lines). A- full calibration curve; B- magnification of the calibration curve at lower concentration points. Brackets illustrate 95% confidence intervals. Parameters of the regression lines were as follows: NanoDrop [measured] = 1.06×[expected]+3.09; Qubit [measured] = 0.97×[expected]−0.61. Intercept was significantly non-zero for NanoDrop only (95% confidence interval of intercept = 1.9–4.3), consistent with the NanoDrop declared limit of detection of 2 ng/µl. (TIF) [file pone.0062692.s001.tif]
